# Functional traits of a native and an invasive clam of the genus *Ruditapes* occurring in sympatry in a coastal lagoon

**DOI:** 10.1038/s41598-018-34556-8

**Published:** 2018-11-15

**Authors:** Marta Lobão Lopes, Joana Patrício Rodrigues, Daniel Crespo, Marina Dolbeth, Ricardo Calado, Ana Isabel Lillebø

**Affiliations:** 1 Department of Biology & CESAM & ECOMARE, University of Aveiro, Campus Universitário de Santiago, 3810-193 Aveiro, Portugal; 2 Centre for Functional Ecology - CFE, Department of Life Sciences, University of Coimbra, Calçada Martim de Freitas, 3000-456 Coimbra, Portugal; 3 CIIMAR - Interdisciplinary Centre of Marine and Environmental Research, Novo Edifício do Terminal de Cruzeiros do Porto de Leixões, Avenida General Norton de Matos s/n, 4450-208 Matosinhos, Portugal

## Abstract

The main objective of this study was to evaluate the functional traits regarding bioturbation activity and its influence in the nutrient cycling of the native clam species *Ruditapes decussatus* and the invasive species *Ruditapes philippinarum* in Ria de Aveiro lagoon. Presently, these species live in sympatry and the impact of the invasive species was evaluated under controlled microcosmos setting, through combined/manipulated ratios of both species, including monospecific scenarios and a control without bivalves. Bioturbation intensity was measured by maximum, median and mean mix depth of particle redistribution, as well as by Surface Boundary Roughness (SBR), using time-lapse fluorescent sediment profile imaging (f-SPI) analysis, through the use of luminophores. Water nutrient concentrations (NH_4_-N, NO_x_-N and PO_4_-P) were also evaluated. This study showed that there were no significant differences in the maximum, median and mean mix depth of particle redistribution, SBR and water nutrient concentrations between the different ratios of clam species tested. Significant differences were only recorded between the control treatment (no bivalves) and those with bivalves. Thus, according to the present work, in a scenario of potential replacement of the native species by the invasive species, no significant differences are anticipated in short- and long-term regarding the tested functional traits.

## Introduction

Coastal ecosystems, like coastal lagoons are complex ecological and socio-economic systems rich in biodiversity and home to a diverse array of habitats and species. Thus, providing numerous economic and societal benefits. Many of these valuable ecosystems are at risk of being irreversibly damaged by invasive species^1,2^. This pressure threatens the ecological functions of these ecosystems and therefore their provision of ecosystem services. Ria de Aveiro is a shallow coastal lagoon, located in the western Atlantic coast of Portugal, characterized by a wide range of habitats with high ecological importance, natural resources of high economic interest and an important ecological diversity, able to provide a wide range of ecosystem services^3^. Bivalves are among the most important commercial resources exploited in this ecosystem, namely the native species Grooved Carpet Shell clam *Ruditapes decussatus* (Linnaeus, 1758) and, since 2011, the invasive species Manila clam *Ruditapes philippinarum (*Adams & Reeve, 1850). Full description of each species is presented as supplementary material (Supplementary Table S1^4–10^).

*Ruditapes decussatus* plays a key role on shell fishing activities and aquaculture due to its high commercial value, about three times that of *R*. *philippinarum*^11^. In 2012, the national aquaculture production of *R*. *decussatus* was of 2 320 tonnes^11^, being Ria de Aveiro and Ria Formosa the main production areas for this species^12^. At an European level, during the last decades, recruitment failures and excessive pressure on the capture of juveniles on natural banks and severe clam mortalities lead to an important decrease in *R*. *decussatus* production^13,14^. This situation resulted in the introduction of the non-indigenous *R*. *philippinarum*. This invasive species originates from the Indo-Pacific region was introduced in Europe for shell fishing activities and aquaculture purposes at the beginning of the 1970s^13^. It was initially introduced in France, followed by England, Spain, Italy and Portugal^13,15,16^. In these countries its habitat overlaps with that of *R*. *decussatus*, a species which it successfully out-competes, not only in aquaculture farms but also on natural conditions^17^. In Arcachon Bay (France), Venice Lagoon (Italy) and the western coast of the Cotentin Peninsula (English Channel), the invasive species has clearly supplanted the native *R*. *decussatus*^1,18–20^. In 2016, the European production of *R*. *philippinarum* was almost seven times higher than that of *R*. *decussatus*, corresponding to 5 389 and 35 436 tonnes, respectively^11^.

In Ria de Aveiro, according to a national management oriented project on bivalves, named Gepeto^21^, the abundance and dispersion of *R*. *decussatus* have been decreasing from 2006/7 to 2013. In a sampling campaign carried out in 2013, 17.0% of the biomass of all bivalves captured in Ria de Aveiro was *R*. *philippinarum* and only 1.0% corresponded to *R*. *decussatus*. In less than 2 years the invasive species became the second most important bivalve species in commercial terms in this ecosystem, with populations mostly dominated by adult specimens larger than the minimum catch size (40 mm). Stocks of *R*. *decussatus* are currently depleted and when the yields recorded from previous campaigns are compared, there was an overall decrease of 67.0% in its biomass and 76.6% in its abundance^21^. Although its commercial value is lower when compared with the native clams, *R*. *philippinarum* constitutes more than 90.0% of the yields of the two species in the European scenario^22^.

Beyond their socio-economic importance, bivalves are major bioturbators of the sediment in marine and estuarine environments^23^. One of the consequences of the introduction of non-indigenous species are shifts in the structure and functioning of ecosystems, as similar species may share physiological traits but that does not mean that they will play similar functional roles^24^. Thus, in spite of the similarities between *R*. *decussatus* and *R*. *philippinarum*, the replacement of one species by the other can potentially change ecosystem structure and functioning. Sedimentary geochemical processes are influenced by benthic invertebrates through bioturbation, which consists in the mixing of unconsolidated sediments and particulate materials during foraging, feeding and burrowing activities, as well as in the enhancement of pore water and solute advection during burrow ventilation^25^ which, consequently influences the concentration of oxygen, pH and redox potential^1^, sediment granulometry^26^, macrofauna diversity^27^, bacterial activity and composition^28,29^ and nutrient cycling^30,31^. Nutrient (NH_4_-N, NO_x_-N and PO_4_-P) concentrations in the water are commonly used to understand ecosystem functioning and numerous studies have described the influence of bivalve aquaculture on various components of the environment, including nutrient cycling^32,33^.

The study of bioturbation is therefore crucial to evaluate the importance of key processes in ecosystems^34,35^. An ecosystem structure and function, may suffer significant changes due to the replacement of its native species by invasive species^36^. In order to evaluate the potential consequences of an eventual replacement of a native clam species by an invasive species, the present study evaluated the functional traits regarding bioturbation activity and its influence in the nutrient cycling of two clam species that occur in sympatry in Ria de Aveiro: the native species *R*. *decussatus* and the invasive species *R*. *philippinarum*. The impact of the invasive species was evaluated under controlled microcosmos conditions, through combined/manipulated ratios of *R*. *philippinarum* and *R*. *decussatus* tested for 21 days. Results will be discussed taking into account sediment luminophores displacement and nutrients concentration in the water column due to bioturbation by *R*. *decussatus* and *R*. *philippinarum* under the tested ratios versus the control conditions without bivalves.

## Results

### Bioturbation

Sediment used in the aquaria was classified as muddy fine sand (ϕ = 2) with 16.3% of fines and an average organic matter content of 5.6 ± 0.4%.

**Figure 1:**
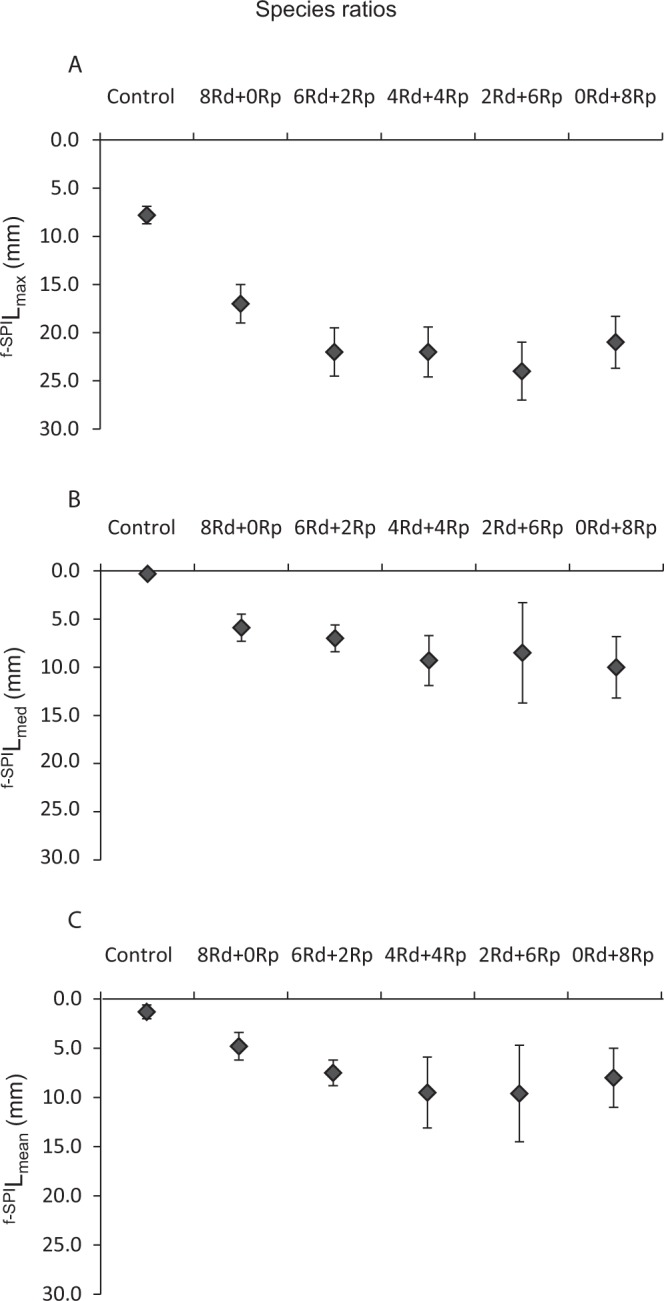
Maximum (^f-SPI^L_max_) (**A**), median (^f-SPI^L_med_) (**B**) and mean (^f-SPI^L_mean_) (**C**) mixed depth of particle redistribution (mm, mean ± SD) in the control (**C**) aquaria and in the aquaria with different treatments of *Ruditapes decussatus* (Rd) and *Ruditapes philippinarum* (Rp) at D_21_.

The ^f-SPI^L_max_ (maximum extent of mixing over the long-term), ^f-SPI^L_med_ (typical short-term depth of mixing) and ^f-SPI^L_mean_ (time-dependent indication of mixing) mixed depth of particle redistribution recorded for the luminophores following the bioturbation process of bivalves, taking into account different ratios of *R*. *decussatus* (Rd) and *R*. *philippinarum* (Rp) tested, are shown in Fig. 1A–C, respectively.

Despite the differences recorded in the ^f-SPI^L_max_ between the different treatments, these were not statistically significant (*pseudo-*F = 1.755; *p* = 0.1782). However, significant differences were found in the ^f-SPI^L_max_ between the control and all the treatments (Table 1). The ^f-SPI^L_max_ recorded for luminophores was lower in the control aquaria (7.8 ± 0.7 mm). Despite there were no significant differences between treatments, the treatment 2 Rd + 6Rp presented the highest value of ^f-SPI^L_max_ (24.0 ± 7.6 mm), while the lowest value corresponded to the treatment only using *R*. *decussatus* (8 Rd + 0Rp) (17.0 ± 3.0 mm). It was possible to observe an increase of the ^f-^^SPI^L_max_ when individuals of both species cohabitated the same aquarium (treatments 6 Rd + 2Rp, 4 Rd + 4Rp, 2 Rd + 6Rp) and with the increasing number of *R*. *philippinarum* (Fig. 1A).

**Table 1:** PERMANOVA main test F values (with associated significance in brackets) between the different treatments of *Ruditapes decussatus* (Rd), *Ruditapes philippinarum* (Rp) (8 Rd + 0Rp, 6 Rd + 2Rp, 4 Rd + 4Rp, 2 Rd + 6Rp, 0 Rd + 8Rp) and between the control (C) and each ratio separately for maximum (^f-SPI^L_max_), median (^f-SPI^L_med_) and mean (^f-SPI^L_mean_) mixed depth of particle redistribution (mm), and SBR (mm) at D_21_. The SBR was not calculated for the control aquaria (n.a).

	^f-SPI^L_max_	^f-SPI^L_med_	^f-SPI^L_mean_	SBR
Ratios	1.755 (0.1782)	1.059 (0.3977)	1.323 (0.2891)	0.830 (0.5237)
C *vs* 8 Rd + 0Rp	57.758 (0.0001)	31.821 (0.0005)	37.941 (0.0004)	n.a.
C *vs* 6 Rd + 2Rp	190.480 (0.0001)	35.330 (0.0003)	47.331 (0.0001)	n.a.
C *vs* 4 Rd + 4Rp	93.790 (0.0001)	40.084 (0.0006)	47.780 (0.0005)	n.a.
C *vs* 2 Rd + 6Rp	43.439 (0.0002)	24.446 (0.0014)	38.309 (0.0005)	n.a.
C *vs* 0 Rd + 8Rp	48.110 (0.0002)	40.947 (0.0003)	48.283 (0.0002)	n.a.

The ^f-SPI^L_med_ between different treatments did not show significant differences (*pseudo-*F = 1.059; *p* = 0.3977) but significant differences were found in the ^f-SPI^L_med_ between the control and all the treatments as is shown in Table 1. The highest and lowest ^f-SPI^L_med_ values (Rp − 10.0 ± 3.2 mm; Rd − 5.9 ± 1.4 mm) were observed in aquaria stocked solely with *R*. *philippinarum* and *R*. *decussatus*, respectively. The control presented lower ^f-SPI^L_med_ values comparatively with the aquaria stocked with bivalves (0.3 ± 0.4 mm). In general, there was a tendency for increase of ^f-SPI^L_med_ with the increasing number of specimens of *R*. *philippinarum* in the treatment (Fig. 1B).

The statistical analysis allowed to test the influence of the ratio of both species on the ^f-SPI^L_mean_ reached by the luminophores, representing the depth in which the organisms were found during most of the study period, and no significant differences were found between treatments (*pseudo-*F = 1.323; *p* = 0.2891). In turn, there were significant differences in the luminophores ^f-SPI^L_mean_ between the control and the aquaria with bivalves (Table 1). Considering the aquaria in which only one species was present (8 Rd + 0Rp; 0 Rd + 8Rp), there was a tendency for the ^f-SPI^L_mean_ values to be lower comparatively with the aquaria in which both species were present (Fig. 1C). Thus, the lowest values of ^f-SPI^L_mean_ were recorded in the monospecific treatment 8 Rd + 0Rp (5.9 ± 1.4 mm) while the highest values were registered in the treatment 2 Rd + 6Rp (10.0 ± 3.2 mm). Considering the total period of the study, aquaria with both species and with highest number of *R*. *philippinarum* tended to reach higher average depths. Control aquaria displayed lower ^f-SPI^L_mean_ values than those with bivalves.

**Figure 2:**
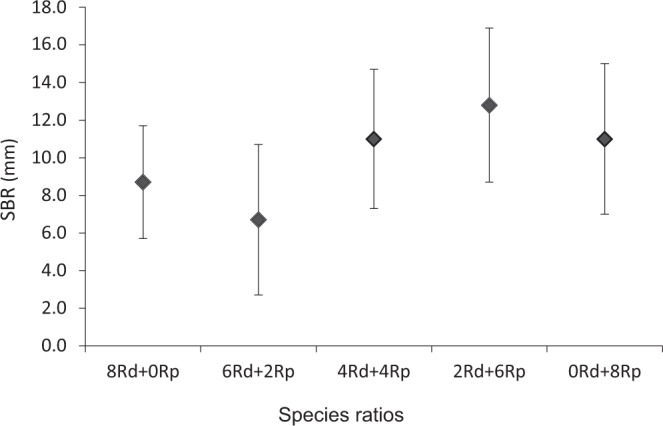
Surface boundary roughness (SBR; mm, mean ± SD) considering different treatments of *Ruditapes decussatus* (Rd) and *Ruditapes philippinarum* (Rp) at D_21_.

The SBR (surface boundary roughness) calculated for the aquaria with bivalves at the end of the experiment is shown in (Fig. 2). The results obtained in the statistical analyses showed no significant differences in the SBR values between the different treatments of *R*. *decussatus* and *R*. *philippinarum* (*pseudo-*F = 0.830; *p* = 0.5237). The average SBR was lower in the aquaria with 6 Rd + 2Rp (7.0 ± 4.0 mm) and higher in the aquaria with the opposite treatment 2 Rd + 6Rp (13.0 ± 4.0 mm). In the treatments with both species, the SBR values tended to increase with the increasing number of *R*. *philippinarum* (Fig. 2).

### Water physicochemical characterization and nutrients concentration

Water temperature, pH, salinity and oxygen concentration of all the aquaria at D_−1_ (day before bivalves’ introduction (D_0_)) and D_21_ (end of the experiment) are shown in Table 2. Temperature ranged from 16.9 ± 0.2 to 17.4 ± 0.2 °C, pH from 7.8 ± 0.3 to 8.2 ± 0.1 and oxygen concentration from 8.5 ± 0.1 to 9.5 ± 0.1 mg L^−1^. Salinity stayed stable at 33 ± 0.0 through D_−1_ to D_21_ in all the aquaria.

**Table 2:** Water temperature (^0^C), pH, salinity and oxygen concentration (mg L^−1^) in the control (C) aquaria and in the aquaria with different ratios of *Ruditapes decussatus* (Rd) and *Ruditapes philippinarum* (Rp) at D_−1_ and D_21_.

	Temperature (^0^C)		pH		Salinity		Oxygen Concentration (mg L^−1^)	
	D_−1_	D_21_	D_−1_	D_21_	D_−1_	D_21_	D_−1_	D_21_
C	16.9 ± 0.1	17.4 ± 0.2	8.2 ± 0.0	8.1 ± 0.0	33 ± 0.0	33 ± 0.0	8.7 ± 0.0	9.5 ± 0.1
8 Rd + 0Rp	16.9 ± 0.1	17.4 ± 0.1	8.2 ± 0.0	7.9 ± 0.0	33 ± 0.0	33 ± 0.0	8.5 ± 0.1	8.7 ± 0.2
6 Rd + 2Rp	16.9 ± 0.1	17.3 ± 0.0	8.1 ± 0.0	7.9 ± 0.0	33 ± 0.0	33 ± 0.0	8.6 ± 0.1	9.0 ± 0.4
4 Rd + 4Rp	16.9 ± 0.1	17.3 ± 0.0	8.1 ± 0.1	7.9 ± 0.1	33 ± 0.0	33 ± 0.0	8.6 ± 0.1	8.9 ± 0.3
2 Rd + 6Rp	16.9 ± 0.2	17.4 ± 0.1	8.2 ± 0.1	7.9 ± 0.1	33 ± 0.0	33 ± 0.0	8.5 ± 0.1	8.9 ± 0.4
0 Rd + 8Rp	16.9 ± 0.2	17.4 ± 0.2	8.1 ± 0.2	7.8 ± 0.3	33 ± 0.0	33 ± 0.0	8.5 ± 0.1	8.8 ± 0.1

**Figure 3:**
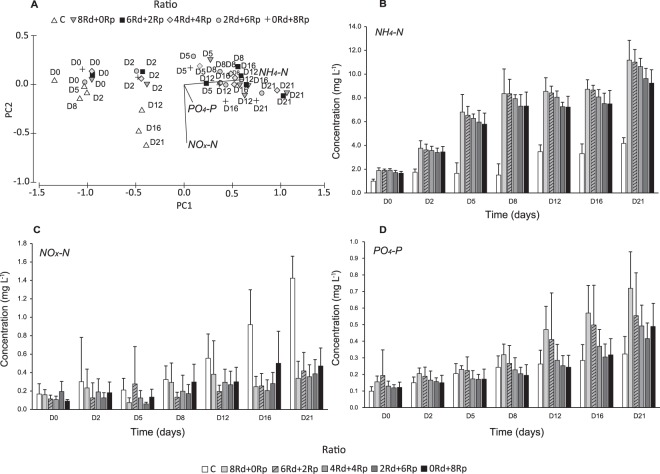
Principal component analysis (**A**) and graphic bars (**B**) based on the average concentrations (mg/L) of ammonium (NH_4_-N), oxidised form of dissolved inorganic nitrogen (NOx-N) and phosphate (PO_4_-P) in the control (**C**) and different treatments of *Ruditapes decussatus* (Rd) and *Ruditapes philippinarum* (Rp) since the beginning until the end of the experiment (D_0_, D_2_, D_5_, D_8_, D_12_, D_16_ and D_21_).

Figure 3A displays the principal components ordination analysis of the average concentration of ammonium (NH_4_-N), oxidised form of dissolved inorganic nitrogen (NO_x_-N) and phosphate (PO_4_-P) in the control aquaria and those stocked with different treatments of *R*. *decussatus* and *R*. *philippinarum* at days 0, 2, 5, 8, 12, 16 and 21. Axis 1 explained 91.0% of the total variance and showed an increasing concentration gradient of NH_4_-N and PO_4_-P from D_0_ to D_21_ in the aquaria with bivalves. Control was mainly characterized by comparatively higher concentrations of NO_x_-N.

Average concentrations of NH_4_-N, NO_x_-N and PO_4_-P in the control aquaria and those stocked with different treatments of *R*. *decussatus* and *R*. *philippinarum* at different sampling days are shown in Fig. 3B–D. The statistical analysis showed that NH_4_-N concentrations were not significantly different between treatments (*pseudo-*F = 2.221; *p* = 0.1016). Significant differences were only found between the control and the aquaria with bivalves (Table 3). In general, NH_4_-N concentrations increased from D_0_ to D_21_, tended to be higher in aquaria with 8 Rd + 0Rp (11.18 mg L^−1^ at D_21_) and decreasing in aquaria with a lower number of individuals of this species (Fig. 3B). In the control, NH_4_-N concentrations were lower, comparatively with the other treatments, achieving a maximum concentration of 3.56 mg L^−1^ at D_21_. In aquaria with 0 Rd + 8Rp the NH_4_-N concentration was lower at the end of the experiment, comparatively with other treatments, reaching a maximum concentration of 9.24 mg L^−1^ (Fig. 3B).

**Table 3:** PERMANOVA main test F values (with associated significance in brackets) between the different treatments of *Ruditapes decussatus* (Rd), *Ruditapes philippinarum* (Rp) (8 Rd + 0Rp, 6 Rd + 2Rp, 4 Rd + 4Rp, 2 Rd + 6Rp, 0 Rd + 8Rp) and between the control (C) and each ratio separately for water nutrients concentration (ammonium (NH_4_-N), oxidised form of dissolved inorganic nitrogen (NO_x_-N = NO_2_-N + NO_3_-N) and phosphate (PO_4_-P) (mg L^−1^)) at D_21_.

	Water Nutrients Concentration		
	NH_4_-N	NO_x_-N	PO_4_-P
Ratios	2.221 (0.1016)	0.358 (0.8326)	1.615 (0.2100)
C *vs* 8 Rd + 0Rp	95.788 (0.0001)	50.463 (0.0003)	11.424 (0.0089)
C *vs* 6 Rd + 2Rp	166.570 (0.0001)	38.570 (0.0003)	3.014 (0.1201)
C *vs* 4 Rd + 4Rp	210.000 (0.0001)	71.633 (0.0001)	3.983 (0.0764)
C *vs* 2 Rd + 6Rp	128.910 (0.0001)	58.611 (0.0001)	1.724 (0.2187)
C *vs* 0 Rd + 8Rp	87.023 (0.0001)	36.277 (0.0004)	3.615 (0.0974)

For NO_x_-N, significant differences were only found in their concentrations at D_21_ between the control and the aquaria with different treatments (Table 3). Between different treatments, concentrations of NO_x_-N were not significantly different (*pseudo-*F = 0.358; *p* = 0.8326). As is shown in Fig. 3C, the average concentration of NO_x_-N was higher in the control than in the aquaria with bivalves, increasing from 0.17 mg L^−1^ at D_0_ to 1.42 mg L^−1^ at D_21_. In the aquaria with bivalves, NO_x_-N concentrations did not show any particular trend for none of the bivalves’ ratios (Fig. 3C).

The statistical analysis of PO_4_-P concentrations showed that there were no significant differences in PO_4_-P concentrations between treatments (*pseudo-*F = 1.615; *p* = 0.2100) but there were significant differences in PO_4_-P concentrations between the control and the aquaria with different treatments (Table 3). The average concentrations of PO_4_-P tended to increase along time, with the control aquaria showing the lowest concentrations that ranged from 0.10 mg L^−1^ at D_0_ to 0.32 at D_21_ (Fig. 3D). At the beginning of the experiment, PO_4_-P concentrations for each ratio were of 0.16 mg L^−1^ for 8 Rd + 0Rp, 0.19 mg L^−1^ for 6 Rd + 2Rp, 0.13 mg L^−1^ for 4 Rd + 4Rp and 0.12 mg L^−1^ for treatments 2 Rd + 6Rp and 0 Rd + 8Rp. At the end of the experiment, PO_4_-P concentrations were higher in the aquaria with higher number of specimens of *R*. *decussatus* (8 Rd + 0Rp − 0.72 mg L^−1^ and 6 Rd + 2Rp − 0.55 mg L^−1^) decreasing in the aquaria with equal or higher number of *R*. *philippinarum* (4 Rd + 4Rp − 0.49 mg L^−1^; 2 Rd + 6Rp − 0.42 mg L^−1^; 0 Rd + 8Rp − 0.49 mg L^−1^).

## Discussion

Bivalves are key species in many habitats due to their influence on habitat engineering and nutrient cycling. While the borrow depth of clams differs from species to species, in general, clams could be considered as shallow-burrowers rather than deep-burrowers, as they tend to preferentially move along and bioturbate the uppermost sediment layers^23^. The depths considered in this study correspond to the depth of the sediment that clams remobilize due to their bioturbation process, being an indicator of their intervention in the ecosystems structure^1^. Results obtained in this study allowed to understand the remobilization behaviour of both clam species, suggesting that, in the short term (^f-SPI^L_med_), *Ruditapes philippinarum* seems to contribute more to sediments remobilization in the superficial layer. At long-term (^f-SPI^L_max_), and when both species occur in sympatry, clams can reach greater depths due to their highest bioturbation intensity. In addition, this trend also occurred in the mean depth (^f-SPI^L_mean_), since the depth at which clams were found in the sediment, during most part of the study period, was greater in aquaria with both species and with higher number of individuals of the invasive species R. *philipinarum*. This finding may be explained by the fact that specimens of *R*. *decussatus* can bury up to 120 mm deep^6^ while those of *R*. *philippinarum* can only reach depths of 80 mm^7^, thus indicating that the native species has the ability to bury at a greater depth. In this way, it is possible that, in the presence of higher densities of *R*. *philippinarum* the native species tends to bury deeper in order to avoid the invasive species. According to Sobral^37^, the immediate response of *R*. *decussatus* to a physical stimuli of sediment movement is a decline in the clearance rate and even the periodic valve closure, which reduces the ability of individuals to maintain their feeding activity and physiological condition, reinforcing the effect that the invasive species can have in the native clam species. However, according to our results, the remobilization depths reached by both species, resulting from their bioturbation activity, were not statistically different. Nonetheless, both species produce a significant effect on sediment remobilization, evidenced by the existence of significant differences between the control aquaria with no bivalves and those stocked with clams. This trend is in accordance with the studies by Sgro *et al*.^38^ and Spencer *et al*.^39^, in which the amount of remobilized sediment was higher in areas exposed to clams bioturbation, revealing that areas with higher densities of clams are characterized by more unstable sediments. The biological modifications carried out by bioturbator organisms increase water-sediment interface roughness and decrease the physical stability of the sediment, which may induce stress in other organisms^40^. In this way, the analysis of the surface boundary roughness (SBR) contributes to the identification of the bioturbation impacts on surface sediment. High values of SBR, resulting from increased fluid transport by the bioturbation activity near the water-sediment interface, reflect a critical zone in which intense physical and mechanical reactions of sediment changes (diagenesis) are promoted^41^. However, according to the results obtained in this study, there were no significant differences in SBR values between different ratios of *R*. *decussatus* and *R*. *philippinarum*.

In an ecosystem, the existence of a species is conditioned by all the environmental processes that occur there, and its existence or disappearance also influences the ecosystem itself, which reveals the network of complex interactions between organisms and their habitat^42^. High densities of clams affect the environment due to the excretion of nitrogenous compounds and the enrichment of sediment with organic matter (faeces and pseudofaeces)^32,43^. High bivalves’ densities are synonymous of high nutrient cycling rates due to bioturbation, since this process intervenes in the nutrient cycle and carbon flux in sediments^34^. The performance of bioturbation processes by a species is favourable to its survival and to the ecosystem itself, but may become unfavourable and of concern if its intensity inhibits the same process in other species and ultimately threatens their survival through a severe sediment destabilization and resuspension increasing^38^. Nevertheless, in the particular case of this study, considering the ratios of clams tested (8 Rd + 0Rp, 6 Rd + 2Rp, 4 Rd + 4Rp, 2 Rd + 6Rp, 0 Rd + 8Rp), there were no significant differences in water nutrient concentrations.

Water concentrations of NH_4_-N and PO_4_-P registered in this study, carried out in microcosms under controlled conditions, were much higher than those observed in natural conditions in Ria de Aveiro. According to Lopes *et al*.^44^, in a study carried out in 2001 and 2012 in Ria de Aveiro, the highest concentrations of NH_4_-N registered were of 0.15 mg L^−1^ in winter 2001 and 0.05 mg L^−1^ in summer 2012, and the highest PO_4_-P concentrations were registered in winter 2001 (0.09 mg L^−1^) and in summer 2012 (0.04 mg L^−1^). Differences in NH_4_-N and PO_4_-P concentrations between microcosms and natural conditions could be explained by the fact that, the sediment was rich in organic matter (5.6%) and that during the experimental period, water in the aquariums was not replaced, thus contributing for a potential build-up of nutrients due to mineralisation processes^30^. In the case of natural conditions, water is daily renewed. The main forcing agent driving water circulation in the lagoon is the tide, which is mesotidal and characterized by semidiurnal tides, presenting an average tidal amplitude at the inlet of 2 m, and amplitudes of 0.6 m in neap tides and 3.2 m in spring tides^45^. Despite the highest concentrations of NH_4_-N and PO_4_-P, no mortality was registered, revealing the capacity of tolerance of both species to high concentrations of these nutrients.

However, the same was not observed in Ria Formosa (south Portugal). In this case, a high mortality of *R*. *decussatus* was registered especially in the intertidal mudflats, which could be related with high summer temperatures at low tide^46,47^, resulting in the accumulation of ammonia in the sediment surface due to the enhancement of mineralization. In fact, Falcão & Vale^48^ found a sharp release rate of ammonium from sediment in the presence of *R*. *decussatus* in Ria Formosa. In this scenario, when tide rises and *R*. *decussatus* starts to feed, this species is exposed to high concentrations of ammonium (>1.40 mg L^−1^) which will decrease with the mixing in the sediment-water interface. The fact that this species exhibits high mortality rates in Ria Formosa when exposed to much lower ammonium concentrations than those observed in the microcosm assay, where no mortality was registered, could be related with the stress to which organisms are exposed during low tide (higher temperature and, consequently, higher salinity). In our study, specimens were always under the same salinity and temperature conditions and always experiencing a “high tide scenario”, as tide was not simulated in the microcosm. Other study, developed by Bartoli *et al*.^49^ in Sacca di Goro shallow coastal lagoon in Italy, showed that *R*. *philippinarum* excrete large quantities of ammonium, which may represent about 90.0% of the total DIN released from sediments with clams, as compared to only 30.0% of the total DIN released from sediments without clams. These results are in accordance with the results obtained in this study in which NH4-N concentrations were not significantly different between treatments but were significantly different between the control and the aquaria with bivalves.

In what concerns to PO_4_-P, it is known that sediments act as a sink or source of phosphate, being sediment physicochemical properties (e.g. granulometry, organic matter content, sediments exchangeable P content, salinity), water temperature and hydrodynamics the main environmental variables influencing the fluxes between sediment and water column^50,51^. Thus, the highest concentrations of PO_4_-P in the aquaria with higher densities of *R*. *decussatus* could be explained by its lower sediment remobilization. Lower hydrodynamics, promotes a higher settlement of organic rich fine particles which adsorb phosphate and decrease the vertical exchanges with the water column (e.g. Mortimer *et al*.^52^, Lillebø *et al*.^53^).

Contrarily to other nutrients, NO_x_-N concentrations in the microcosm (ranged from 0.34 mg L^−1^ to 1.42 mg L^−1^) were in the same range to that observed in Ria de Aveiro in 2001 and 2012 (ranged from 0.01 mg L^−1^ to 2.33 mg L^−1^) (personal info.). Bivalves’ bioturbation minimizes the potential negative effects of excessive biodeposition, as particle and solute transport by bioturbation significantly influences rates and pathways of organic matter mineralization, particularly by increasing oxygen penetration depth in sediments. Thus, contrarily to what was observed, it was expected that NO_x_-N concentrations in treatments with bivalves were higher than those in the control^52^. In the presence of bivalves, sediment was more aerated due to a higher remobilization, thus enabling the bacterial activity in the sediment, mediating nitrification by oxidising NH_4_ to NO_x_, using O_2_.

Overall, this study allowed to conclude that, based on the ratios tested, will not occur significant changes in sediment bioturbation, at short- and also at long-term, and nutrient cycling in a scenario of potential replacement of a native and an invasive clam of the genus *Ruditapes* occurring in sympatry in a coastal lagoon.

## Material and Methods

### Natural ecosystem under study

Ria de Aveiro is located on the northwestern coast of Portugal, between 40°38′N and 40^o^ 57′N. This temperate system is a shallow coastal lagoon characterized by intertidal mud and sand flats, salt marshes and islands, forming four main channels, Mira, Ílhavo, Espinheiro and S. Jacinto-Ovar. The only connection with the Atlantic Ocean is made through an artificial inlet constructed in 1808 (1.3 km length; 350 m wide; 20 m depth). Ria de Aveiro is mesotidal, characterized by semidiurnal tides. It exhibits a longitudinal gradient of salinity from about 0 at upstream areas, to about 36 at the Ocean boundary.

### Microcosm setting

The microcosm set-up was designed to simulate different degrees of sympatry occurrence in Ria de Aveiro of a native and an invasive clam of the genus *Ruditapes*, and run with specimens of *R*. *decussatus* (Rd) and *R*. *philippinarum* (Rp) from the lagoon. Taking into account the large number of specimens necessary, the convenience of being of the same size and the effort associated with their capture, post-depuration clams were acquired in the local depuration centre that receives the specimens collected by local fishermen. Shell length of the individuals used in this experiment was measured with a Vernier calliper to the nearest 0.05 mm. The average length was of 44.8 ± 1.8 mm for Rd and of 41.8 ± 2.3 mm for Rp. Specimens were transported to the laboratory and placed in containers with synthetic saltwater (prepared by mixing Red Sea Coral Pro Salt (Germany) and freshwater purified by a reverse osmosis unit) without sediment, with a salinity of 33 during 72 hours. This salinity was stablished based on the distribution areas of both species in Ria the Aveiro^21,54,55^. No food was added during acclimation and continuous aeration was kept by air-bubbling the water with air-stones. The sediment used in the experiment was collected in Mira channel, homogenized and resident macrofauna was visually detected and removed. Five sediment sub-samples were analysed to determine grain-size and organic matter content. Sediment was distributed along 30 glass aquaria (150 × 150 × 500 mm), creating a 150 mm column about 1/3 of the height of the aquarium and filled with saltwater (about 2/3 of the height of the aquarium) and left to settle for 48 h. Then, all saltwater was replaced and left to stabilize for 24 h before the introduction of bivalves. Aquaria were gently aerated by air-bubbling using an air pump (Aqua Medic Mistral 4000, 4000 l/h) connected to a silicon tube (ø = 4.00 mm) in which were connected silicon capillary tubes (ø = 0.84 mm) that aerated each aquarium. Aquaria were randomly distributed by the bench and exposed to natural light conditions with a 10 h day: 14 h dark photoperiod. Specimens of Rd and Rp were placed in the aquaria at different ratios (8 Rd + 0Rp, 6 Rd + 2Rp, 4 Rd + 4Rp, 2 Rd + 6Rp, 0 Rd + 8Rp), with 5 replicates per each one of them, totalling 25 aquaria. Five aquaria were kept without bivalves (0 Rd + 0Rp) and used as control. All aquaria were covered with parafilm to minimize evaporation and hence pronounced salinity shifts. In order to avoid the initial activity associated with bivalves establishment and borrowing, 40 g of fluorescent-dyed sediment particles (luminophores: 125–250 µm diameter, pink colour; Brian Clegg Ltd., UK) were distributed in the front face of each aquaria 24 h after the introduction of bivalves (D_0_). The same quantity of luminophores was added to all the aquaria during the experiment at days 0, 4, 11 and 17 to allow the monitoring of bioturbation. The water of each container was not renewed and neither sediment nor food were added. The temperature in the room ranged from 16.4 °C at D_−1_ to 17.9 °C at D_21_.

### Bioturbation

**Figure 4:**
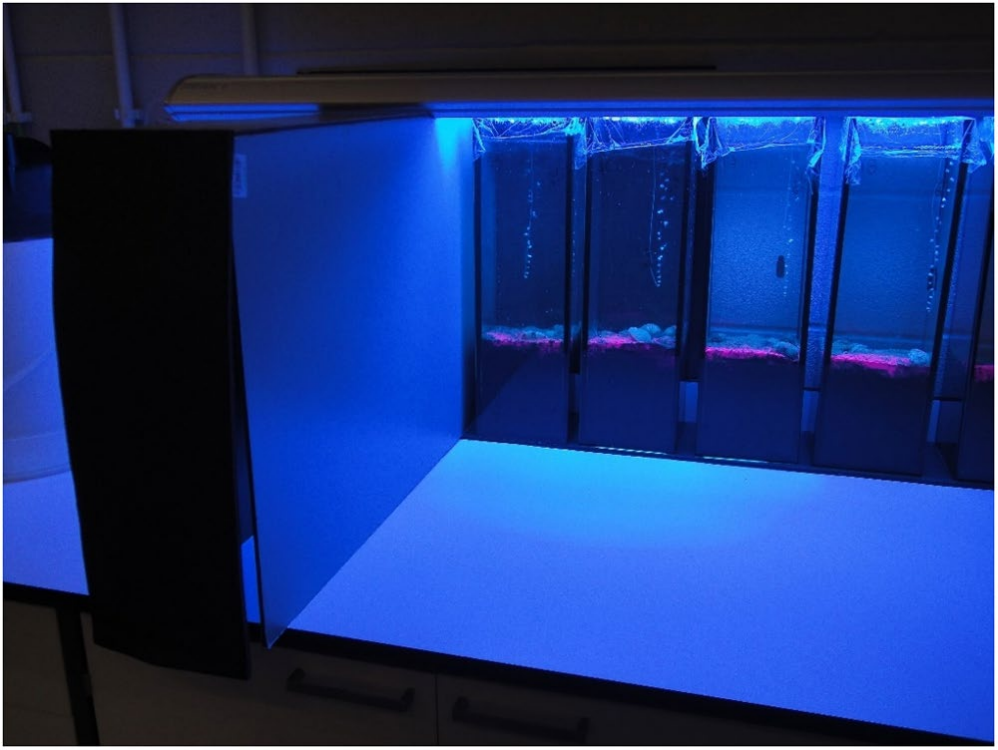
Particle reworking was measured inside a black box, photographing the pink luminophores exposed to four actinic lights 22000 K Rede-sea 80 W (λ = 400–450 nm).

**Figure 5:**
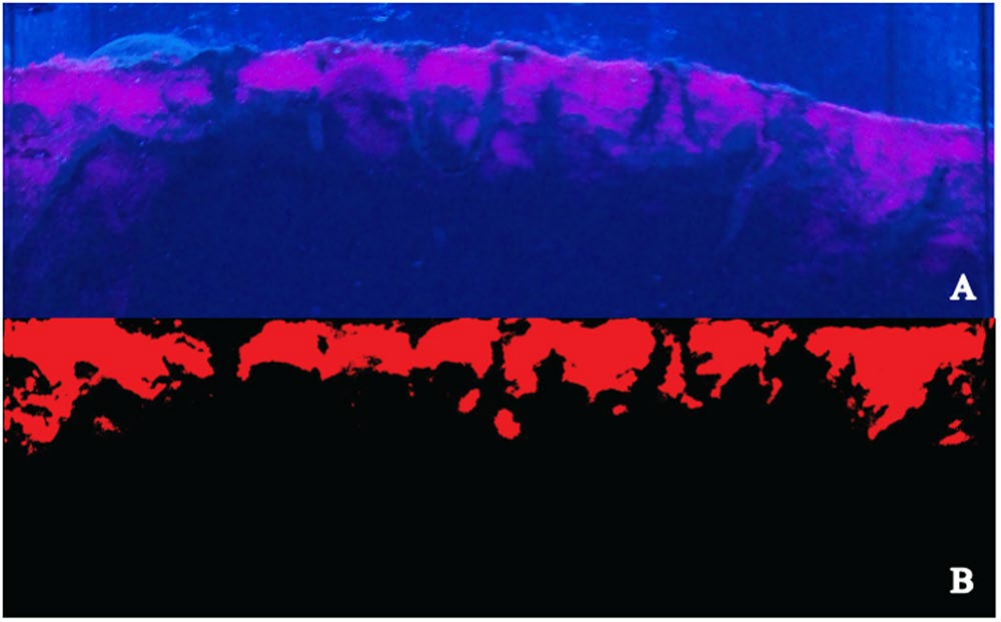
Image analysis for the quantification of luminophores showing (**A**) the original image with the sediment appearing in black and luminophore particles in pink, and (**B**) the processed image, showing the flattened sediment-water interface.

Particle reworking was measured non-invasively using fluorescent sediment profile imaging (f-SPI^56^) and luminophores. To photograph the aquaria, a black box was used, illuminated with four actinic fluorescent lamps (22.000 K, 80 W (λ = 400–450 nm), Red-Sea) (Fig. 4). Images were captured once a day, using a SONY Cyber-shot G (14MP; aperture f = 5.6) camera. Considering the main objective of this study, solely the images collected at D_21_ were analysed. Images obtained were previously cut according to the internal width of the aquarium (1968 pixels; effective resolution = 0.07 mm per pixel) and then converted to a red-blue-green (RGB) stack and saved with JPEG compression (Joint Photographic Experts Group (Fig. 5)). Images were then analysed using a custom-made plugin that runs within ImageJ (Version 1.48c), a java-based public domain program developed at the US National Institutes of Health (available at http://rsb.info.nih.gov/ij/index.html). In the particular case of this study, as we added luminophores several times along the experiment, in the control aquaria, luminophores created a superficial layer which was not remobilized. For this reason, in the control aquaria the interface considered was between the sediment and the layer of luminophores. As all luminophores were remobilized in the aquaria stocked with bivalves, the sediment-water interface was considered for analysis. At D_21_ the following parameters were determined: mean (^f-SPI^L_mean_, time-dependent indication of mixing), median (^f-SPI^L_med_, typical short-term depth of mixing), and maximum (^f-SPI^L_max_, maximum extent of mixing over the long-term) mixed depth of particle redistribution, following Hale *et al*.^57^. In addition, the maximum vertical deviation of the sediment-water interface (upper-lower limit = surface boundary roughness, SBR), which provides an indication of surficial faunal activity, was also determined. SBR was only calculated for aquaria stocked with bivalves, as these were the only ones in which there was a sediment-water interface remobilization.

Sediment grain-size was analysed by dry sieving following Dias^58^. Median and percentage of fines were used to classify the sediment, according to the Wentworth scale^59,60^. Total organic matter concentration was obtained as described by Kristensen & Anderson^61^.

### Water characteristics and nutrient analyses

Water aliquots (10 ml) were sampled from each aquarium at days 0, 2, 5, 8, 12, 16 and 21 (the volume of water sampled throughout the experiment represented less than 10.0% of the microcosm total volume). Samples were filtered through Whatman GF/C glass-fibre filters and stored at −20 °C until analysis of dissolved inorganic nutrients (ammonium, NH_4_-N; oxidised form of dissolved inorganic nitrogen, NO_x_-N; phosphate, PO_4_-P). The determination of the concentrations of NH_4_-N and PO_4_-P was performed following standard methods described in Limnologisk Metodik^62^. The concentrations of NO_x_-N were determined using a flow injection system (FIAstar 5000 Analyzer, Höganäs, Sweden), following the Strickland & Parsons^63^ method. To ensure analytical quality control, calibration curves, using a standard solution, were run at the beginning of the analysis and in parallel with blanks and samples. Water temperature, pH, concentration of dissolved oxygen and salinity were measured using a WTW – pH 330i/set equipped with SenTix® 41; a WTW – cond 3110/set 1 equipped with TetraCon® 325 and a WTW – Oxi 3210/set 2 equipped with CellOx® 325-3.

### Statistical analyses

Bioturbation activity and water nutrient concentrations were analysed using a model with one fixed factor (clam ratios) for D_21_. Aquaria without bivalves were used as a control for analysis, with the exception of the SBR variable, as there was no vertical variation on the superficial layer due to the absence of organisms. The resemblance matrix between samples was obtained using Euclidean distances following a Log(X + 1) transformation. The ^f-SPI^L_max_ data were analysed under the null hypothesis (H_0_) of no significant differences in the maximum extent of luminophores mixing in the sediment over the long-term considering different treatments of *R*. *decussatus* (Rd) and *R*. *philippinarum* (Rp). Data concerning ^f-SPI^L_med_ was analysed under the H_0_ of no significant differences in the luminophores mixing in the sediment over the short-term, considering different treatments of Rd and Rp. Concerning ^f-SPI^L_mean_ data, this was analysed under the H_0_ of no significant differences in the average depth reached by luminophores in the sediment, considering different treatments of Rd and Rp. The SBR analysis was performed under the H_0_ of no significant differences recorded in SBR determined considering different treatments of Rd and Rp. Water nutrient concentrations was analysed under the H_0_ of no significant differences in the water nutrient concentrations (NH_4_-N, NO_x_-N and PO_4_-P, separately) considering different treatments of Rd and Rp. All H_0_ detailed above were tested between the control aquaria and different treatments of Rd and Rp for all the variables previously described, with the exception of SBR. Statistical differences between treatments were tested based on a matrix only with data from aquaria with bivalves, excluding the control. The comparison between control aquaria and those stocked with different treatments of clams was performed separately for each clam treatment. Hypothesis testing was performed by Permutation Multivariate Analysis of Variance^64^, using the software PRIMER v6^65^, with the add-on PERMANOVA+^66^. To run the PERMANOVA tests, we considered 9999 Monte Carlo permutations. The pseudo-*F* values in the main tests and the *t*-statistic in the pairwise comparisons were evaluated in terms of the significance among levels of the tested factor. Values of *p* < 0.05 revealed that the groups differed significantly. Water nutrient concentrations of NH_4_-N, NO_x_-N and PO_4_-P were represented in ordination analyses, using a Principal Component Analysis (PCA)^64^.

## Electronic supplementary material

Table S1
