# Supplementary material for: Functional traits of a native and an invasive clam of the genus Ruditapes occurring in sympatry in a coastal lagoon
Source: Sci Rep. 2018 Nov 15;8:16901. doi: 10.1038/s41598-018-34556-8 (PMC6237838; doi:10.1038/s41598-018-34556-8)
Supplement: Supplementary file 1 — Table S1 [file 41598_2018_34556_MOESM1_ESM.pdf]

Functional traits of a native and an invasive clam of the genus *Ruditapes* occurring in sympatry in a coastal lagoon

Marta Lobão Lopes[1], Joana Patrício Rodrigues[1], Daniel Crespo[2], Marina Dolbeth[1,3], Ricardo Calado[1], Ana Isabel Lillebø[1]

[1]Department of Biology & CESAM & ECOMARE, University of Aveiro, Campus Universitário de Santiago, 3810-193 Aveiro, Portugal. [2]Centre for Functional Ecology - CFE, Department of Life Sciences, University of Coimbra, Calçada Martim de Freitas, 3000-456, Coimbra, Portugal. [3]CIIMAR - Interdisciplinary Centre of Marine and Environmental Research, Novo Edifício do Terminal de Cruzeiros do Porto de Leixões, Avenida General Norton de Matos s/n, 4450-208, Matosinhos, Portugal. Correspondence and requests for materials should be addressed to M.L.L. (email: martalopes@ua.pt) and A.I.L. (email: lillebø@ua.pt).

Table S1. Description of the diagnostic features, geographic distribution, habitat, feeding strategy and reproduction of *Ruditapes decussatus* and *Ruditapes philippinarum*.

**Scientific Name**	Ruditapes decussatus (Linnaeus, 1758)	*Ruditapes philippinarum* (Adams & Reeve, 1850)
**Common Name**	Grooved Carpet Shell clam	Manila clam
**Diagnostic Features**	Shell broadly oval to quadrate, umbones distinctly anterior. Posterior hinge line straight, posterior margin truncate, anterior hinge line grading into the down-sloping anterior margin. Posteriorly, the shell is conspicuously decussate and prominent. Sculpture of fine concentric striate and bolder radiating lines. Growth stages clear. Lunule and escutcheon poorly defined. Three cardinal teeth in each valve, the central one in left valve and the others (bifid) in right. Pallial line and adductor scars distinct. The pallial sinus presents U-shaped, not extending beyond mid-line of shell, but reaching a point below the posterior part of the ligament. Lower limb of sinus distinct from pallial line for the whole of its length. Inner surfaces glossy white, often with yellow or orange tints, and with a bluish tinge along dorsal edge. Its colour varies (cream, yellowish or light brown), often with darker markings. Long siphons completely separated and little marked radial striate are the main characteristics differentiating this species from *R. philippinarum*. Length up to 75 mm[4].	Shell equivalve, inequilateral, somewhat broadly oval in outline. Lunule elongate heart-shaped, not particularly well defined and with light and dark brown fine radiating ridges. Sculpture of radiating ribs and concentric grooves, the latter becoming particularly sharp over the anterior and posterior parts of the shell, making the surface pronounced decussate. Growth stages clear. Three cardinal teeth in each valve, the central one in left valve and the others (bifid) in right. Pallial sinus not extending beyond the centre of the shell, leaving a wedge-shaped space between its lower limb and the pallial line. Colour and pattern variable (white, yellow or light brown, sometimes with rays, steaks, blotches or zig-zags of a darker brown, slightly polished). Inside of shell polished white with an orange tint, sometimes with purple over a wide area below the umbones. Similar to *R. decussatus* but differs from it by the much more pronounced dessate sculpture and colour; and smaller siphons not completely separated. Maximum length 80 mm[5].

**Habitat**	Sand, muddy gravel or clay, usually in quiet waters. Lower shore and shallow subtidal. This species can burrowed up to 100-120 mm depth[4,6,7].	Sand, muddy gravel or stiff clay in intertidal and subtidal areas of estuaries and coastal lagoons. It is a shallow burrower, commonly found between 40-80 mm depth[5,7,8].
**Geographical Distribution**	From southern and western England to the Iberian Peninsula and into the Mediterranean. South to western Morocco and Senegal, west Africa[9].	*R. philippinarum* is native to Japan with a wide distribution in the Indian and Pacific Oceans from Pakistan to the Russian Federation (Kuril Islands). It has been introduced along the north American Pacific coast, the Hawaiian Isles and along the European coastline from the United Kingdom to the Mediterranean Basin[5].
**Feeding Strategy**	Suspension-feeding. It filters water through its two siphons (one in and the other out) catching organic matter (detritus) and phytoplankton[4,6.]	Suspension-feeding. It filters water through its two siphons (one in and the other out) catching organic matter (detritus) and phytoplankton[5,10].
**Reproduction**	Sexes are separate, although hermaphrodites can be found. Reproduction is external and takes places mainly during summer. In spring, clams can be artificially conditioned for hatching with higher temperature water and abundant food. Larvae swim freely for 10-15 days before settling as spat of about 0.5 mm on a sand and silty mud substrate[4].	Strictly gonochoric. The period of reproduction varies according to the geographical area. A period of sexual rest is observed from late autumn to early winter. Gametogenesis lasts 2-5 months, followed by the spawning. A second spawning event may occur in the same season, 2-3 months later. Temperature and feeding are the two main parameters affecting gametogenesis, which can be initiated at 8-10 °C and is accelerated by rising seawater temperature. Although the optimal temperature to spawn efficiently is between 20 and 22 °C, 12 °C is the minimum threshold below which it cannot spawn. Larval development lasts 2 to 4 weeks before spatfall. Settlement size is between 190-235 µm in shell length[5].
